# A Life-Threatening Case of Torsemide-Induced Toxic Epidermal Necrolysis Associated With the Treatment of Anasarca

**DOI:** 10.7759/cureus.22895

**Published:** 2022-03-06

**Authors:** Sujana Reddy, Bray K Aron, John Stewart

**Affiliations:** 1 Internal Medicine, East Alabama Health, Opelika, USA; 2 Osteopathic Dermatology, East Alabama Health, Opelika, USA; 3 Dermatology, East Alabama Health, Opelika, USA

**Keywords:** alcoholic cirrhosis, torsemide, type iv hypersensitivity, medication allergy, sulfa drug, toxic epidermal necrolysis (ten), stevens-johnson syndrome (sjs)

## Abstract

Toxic Epidermal Necrolysis (TEN), along with Stevens-Johnson Syndrome (SJS), are rare, life-threatening delayed type IV hypersensitivity mucocutaneous skin disorders that can often be precipitated by medications. The most common culprits are sulfonamide antibiotics and various antiseizure medications. We report a case of a 41-year-old Black female that initially presented with SJS, which then rapidly progressed to TEN, confirmed by hematoxylin and eosin stain skin biopsies. Approximately 80% of her body surface area had necrosis and epidermal detachment lesions. It was concluded that TEN was caused by the use of torsemide for treatment of her underlying diffuse anasarca attributable to alcoholic cirrhosis. During her one-month hospital stay, a multi-disciplinary team consisting of dermatology, gynecology, rheumatology, nephrology, and infectious disease evaluated and treated the patient. Interventions included various supportive care measures as well as intravenous steroids, cyclosporine, plasma exchange, and intravenous immunoglobulin. Given that the mortality rate for TEN is over 30%, and this patient had end-stage cirrhosis, her prognosis was extremely poor. Even though her TEN eventually healed slowly, the patient experienced complications. This case demonstrates the importance of cautiously using sulfonamide medications in patients with known hypersensitivity to sulfa drugs.

## Introduction

Stevens-Johnson Syndrome (SJS) and Toxic Epidermal Necrolysis (TEN) are severe skin disorders that involve necrosis and detachment of the epidermis. In over 90% of patients, mucous membranes are affected, which includes the mouth, eyes, and the urogenital mucosa. SJS is characterized by skin detachment of less than 10% of the body surface; whereas, TEN is characterized by skin detachment of greater than 30% of the body surface. For patients with skin detachment covering 10-30% of the body surface, it is SJS/TEN overlap [1,2,3]. This is an immunologic response and type IV hypersensitivity reaction where immune complexes form from the causal agent’s metabolites, as the body is unable to detoxify them. Cytotoxic T-cells attack the keratinocytes and epithelial cells leading to cell death and sloughing of the skin [1,3,4]. 

SJS and TEN both start with a prodrome of fever, generalized malaise, and/or muscle aches. Early signs of mucosal involvement may be signaled by pain while swallowing or eye discomfort. Following the prodrome is the development of painful, coalescing erythematous macules with purpuric centers. Usually, this rash starts on the thorax or face before spreading elsewhere. Within a few days, vesicles and bullae develop, and sloughing of the skin occurs [4]. Progression to painful erosions and hemorrhagic crusting on the lips and vermillion border as well as inside the mouth can occur. Ocular involvement usually manifests as severe conjunctivitis with purulent discharge; however, it is important to note that corneal involvement can also be involved in cases. For urogenital involvement, genital erosions and urethritis are common manifestations [4,5,6]. 

Patients that are at a higher risk for SJS/TEN include those with immunocompromised states such as malignancy or HIV and epilepsy patients. These patients are at a higher risk because the medications they are taking often are known to cause SJS and TEN [5]. Common medications known to cause these skin disorders are allopurinol, aromatic antiseizure medications, lamotrigine, antibacterial sulfonamides, nevirapine, oxicam nonsteroidal anti-inflammatory drugs, and others [3,4]. However, in approximately 20% of patients, no trigger can be identified [6]. A large case-control study looked at the association between commonly prescribed drugs and SJS/TEN. It found that sulfonamides, specifically antibacterial sulfonamides, had the highest associated risk for SJS and TEN within the first few weeks of use and with only one dose of the medication. Although structurally related, sulfonamide diuretics and sulfonylureas were not associated with increased risks [2,7]. Torsemide is a non-antibacterial sulfonamide. Non-antibacterial sulfonamides have been less frequently associated with causing SJS and TEN. Torsemide-induced TEN is an exceptional finding. To the best of our knowledge, only one case of TEN has been reported in association with torsemide [8]. Moreover, only one other report of a non-antibacterial sulfonamide diuretic-induced SJS/TEN has ever been documented [6,8,9]. We report the second case of torsemide-induced TEN. 

## Case presentation

A 41-year-old Black female, with a past medical history of alcoholic cirrhosis, anemia, gastroesophageal reflux disease, hypertension, and amenorrhea, who was recently discharged from the hospital, came back to the emergency room with a chief complaint of a “diffuse rash.” During her most recent 11-day hospitalization, the patient was diagnosed with alcoholic cirrhosis and portal hypertension. Numerous varices were seen on computed tomography throughout her spleen, esophagus, and stomach. The patient was already taking home medications of nadolol, methylprednisolone, and pantoprazole. Acute onset of anasarca was the reason for her 11-day hospital stay. She was placed on spironolactone 25 mg twice a day and furosemide 40 mg, both orally, but there was no significant improvement in her swelling and discomfort. The patient was then switched to intravenous furosemide 20 mg twice daily along with 1.5 L of fluid and sodium restriction, which showed promising improvements with roughly a 10-kg weight loss. During that admission, an uncomplicated urinary tract infection was discovered, and the patient completed treatment with ceftriaxone prior to discharge. The patient was transitioned from IV furosemide to torsemide 20 mg orally once a day along with spironolactone 100 mg orally once a day, nadolol 20 mg orally once a day, lactulose 10 gm orally twice a day for seven days, and pantoprazole 40 mg orally once a day at the time of discharge. The patient came back to the emergency room 10 days after discharge where she was diagnosed with an unresolved urinary tract infection. Also noted was a diffuse maculopapular, non-blanching rash that extended to the bottom of her feet and the palms of her hands. Mucous membranes were also noted to have a few subtle lesions. During this ED visit, the rash was attributed to her elevated ammonia level secondary to chronic cirrhosis. She was discharged with a prescription for nitrofurantoin for her urinary tract infection but she never filled or took this medication. 

**Figure 1:**
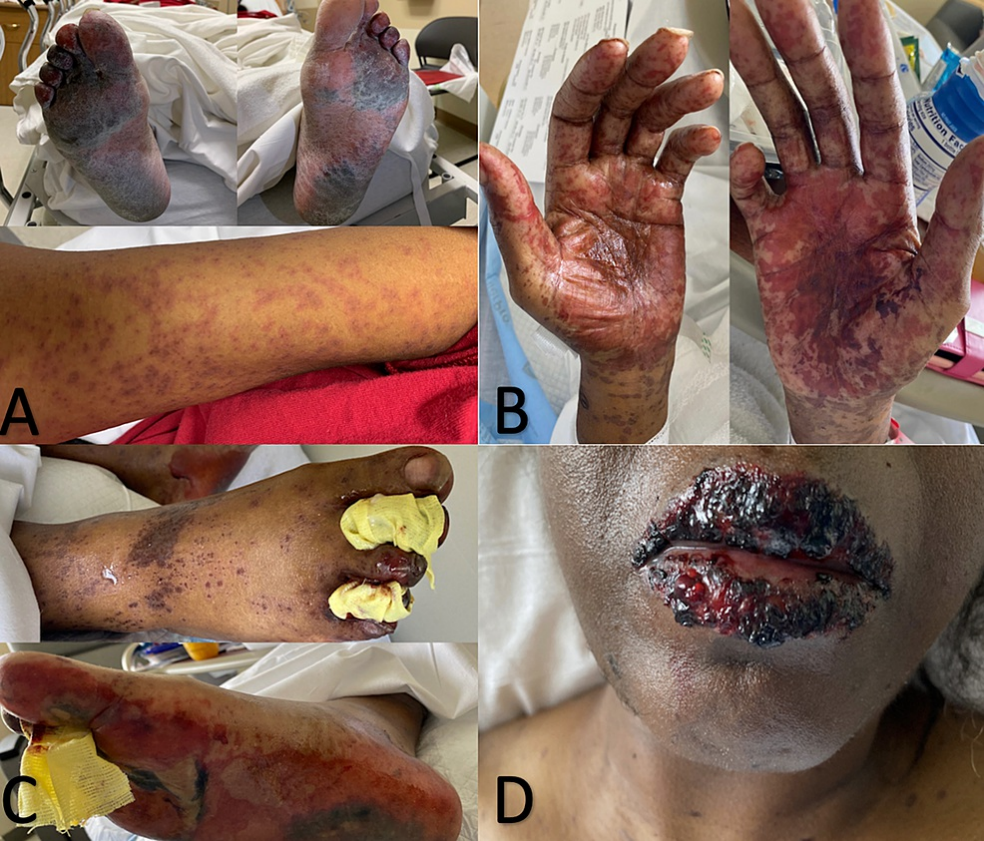
(A) Initial presentation on admission of diffuse rash on the soles of the feet and upper arms; (B) Erythematous macules and blisters on the palmar aspects of the hands; (C) Maculopapular rash on the soles of the feet; (D) Hemorrhagic crusting of the vermillion lips.

The patient was brought back to the emergency room by emergency medical services two days after discharge. The patient presented with generalized weakness and a diffuse rash that was now present on her anterior and posterior arms, hands, and feet (Figure 1A). She also had some involvement of the anterior waistline with small areas of excoriation. The patient stated that this diffuse rash started about four days ago and didn't recall ever developing a rash like this before. Blisters initially appeared on the bottom of her feet and slowly spread to the rest of her body. The patient reported sloughing off of her skin when touched. She was extremely weak and had difficulty ambulating. The rash was painful and pruritic at times. She also noted that her mouth was painful when she ate salty foods. She rated the pain as 9/10, especially on the bottom of her feet. No fever or chills were reported. She did endorse a history of medication allergy to penicillin that caused rash, itching, and generalized edema. On a physical exam, a confluent macular rash was present on her bilateral palms with loss of skin noted to her right and left forearms (Figure 1B). A scattered maculopapular eruption was present on the anterior and posterior torso as well as her bilateral lower extremities. Large bullae were present on the plantar side of her feet (Figure 1C). On admission, C-reactive protein (CRP) was elevated to 3.2 mg/dL, lactic acid was elevated at 2.2 mmol/L, and leukocytes were 11.2 K/uL. All previous medications were discontinued. 

During this admission, a multi-disciplinary team consisting of dermatology, gynecology, rheumatology, nephrology, and infectious disease were consulted to help with care. The patient's rash began to spread and rapidly develop. Some noticeable changes involved: the rash spreading all over her trunk, bleeding over the lips (Figure 1D), buccal mucosa with erythematous papules, bullous changes on the hands, hemorrhagic changes over her abdomen, and denuded skin in the perivaginal and perirectal area. More than 80% of her body was covered in these lesions. A pelvic exam showed that the external genitalia was inflamed bilaterally and vaginal lesions were noted on her left sidewall without blood or exposure to the fascia. Dermatology obtained biopsies from her right lower leg. The patient was treated with NaCl 0.9% 1000mL IV 75mL/hr, Solu-Medrol 125 mg IV twice daily, valacyclovir 500 mg orally every 12 hours, Benadryl 50 mg IV as needed, Vaseline topical ointment three times a day, lidocaine 5% topical ointment three times a day, clobetasol 0.05% topical ointment twice a day, diphenhydramine hydrochloride/lidocaine/aluminum hydroxide/magnesium hydroxide swish and swallow (magic mouthwash) 15 mL three times a day, oxycodone 5 mg IV every four hours, and morphine 2 mg IV every two hours. Per gynecology, intravaginal topical hydrocortisone ointment with a vaginal mold made of a glove and foam rubber was implemented for three days on and four days off. Also, topical betamethasone dipropionate 0.05% ointment was applied externally to the vulva twice a day. The patient was then put on cyclosporine 100 orally every 12 hours.

**Figure 2:**
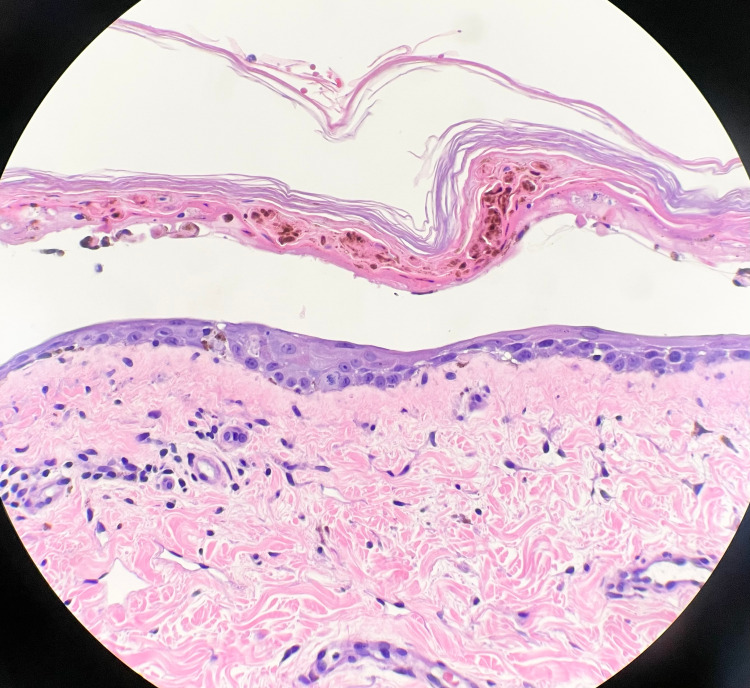
Skin biopsy with hematoxylin & eosin stain showing full-thickness epidermis detachment from dermis and necrosis as well as moderate lymphocyte predominance consistent with TEN. TEN: toxic epidermal necrolysis

Despite IV steroids and cyclosporine, the lesions persisted and the patient was not improving. Rheumatology was consulted to rule out any autoimmune processes. Tests for tuberculosis, HIV antibodies, and hepatitis panel were all negative. Anti-nuclear, anti-smith, anti-double stranded DNA, beta-2 glycoprotein, cardiolipin, scleroderma, lupus anticoagulant, anti-Sjögren's syndrome A/anti-Sjögren's syndrome B (SSA/SSB) antibodies were all negative. Inflammatory markers were elevated with a CRP of 3.5 mg/dL and sedimentation rate of 77 mm/hr. Antihistone antibodies were positive at 4.2 units. The patient did have low complements with component 3 (C3) at 65 mg/dL and component 4 (C4) <8 mg/dL, but it was believed to be low due to liver failure. Rheumatology overall excluded any autoimmune pathologies. Considering her severe liver disease, it was concluded that the possible culprit of her lesions was torsemide as it has hepatic metabolism in 80% of people via the CYP2C9 pathway. Nephrology evaluated and treated the patient with 3L plasma exchange to remove any significant burden of remaining medication or toxin. A right internal jugular vascular access catheter (Vas Cath) was placed by surgery for the plasma exchange treatment. The patient then was given 70 g of intravenous immune globulin (IVIG) over five days. Biopsy results returned and two biopsies for hematoxylin and eosin stains were positive for TEN, while the one biopsy for direct immunofluorescence came back negative (Figure 2). The patient developed hypertension and increasing anasarca and so was started on amlodipine 5 mg once daily, amiloride 5 mg twice daily, and ethacrynic acid 50 mg once a day as this was not a sulfa drug. 

The Wound Care physician followed and noted that her diffuse body lesions were slowly improving over the course of her stay. During the hospitalization, her ammonia level increased and she became encephalopathic; therefore, she was started on lactulose 20 gm syrup with rectal tube placement to prevent infection given open lesions at the rectum. Her course was further complicated as she later developed sepsis with methicillin-susceptible *Staphylococcus aureus* (MSSA) bacteremia from the Vas Cath that was placed for plasmapheresis. The Vas Cath was removed and she was initially started on 1250 mg vancomycin IV every eight hours, which was discontinued to cefazolin 2 g every eight hours for 14 days by the Infectious Disease specialist. A lumbar MRI was done to rule out any osteomyelitis or abscess and a transesophageal echocardiogram (TEE) to rule out endocarditis. Both lumbar MRI and TEE were negative. Throughout her stay, the patient required multiple blood transfusions, secondary to her varices. Surgery was consulted but did not perform endoscopy as the patient was a high risk given all her complications thus far. The patient was then transferred to a comprehensive rehab center, which included physical therapy/occupational therapy/speech therapy (PT/OT/ST) and wound care with additional treatment of 100 mg doxycycline orally twice daily for 10 days. The patient made good progress during rehab but unfortunately reached a new baseline given her end-stage liver disease. She was discharged home with hospice and ultimately passed away surrounded by her loved ones. 

## Discussion

SJS and TEN are rare and severe mucocutaneous skin conditions that are often secondary to pharmacotherapy [6]. The estimated incidence of TEN is about 0.4-1.2 cases per million people per year with an estimated mortality of 25-30% [2,10]. Differential diagnoses for SJS and TEN include erythema multiforme, staphylococcal scalded skin syndrome, acute generalized exanthematous pustulosis, generalized bullous fixed drug eruption, and phototoxic eruptions [1,2].

Signs of SJS/TEN typically present within 5-28 days after starting drug therapy and in some cases can pose a risk up to three months [8,10]. This case shows how imperative it is to undertake a thorough medication reconciliation history to include a timeline of prescription medications as well as any over-the-counter drugs, herbal supplements, and exposure to recreational drugs [11]. SJS and TEN have commonly been associated with sulfonamide antibiotics [2,3]. A case-controlled study showed sulfonamide antibiotics to be at the highest risk among known drugs to cause SJS and TEN. According to the study, other drugs with increased risk to cause SJS and TEN were anticonvulsant agents, oxicam agents, allopurinol, chlormezanone, and corticosteroids [9]. Nonantibiotic sulfonamides like torsemide have rarely been reported [4]. 

Narrowing down which medication was the etiology of the patient’s TEN condition was a challenge. The patient was questioned multiple times regarding her past medication history and compliance. Proton pump inhibitors (PPI) rarely induce SJS or TEN, and the patient did state given her acid reflux history, she had taken over-the-counter PPI with no adverse reactions. Furosemide was ruled out because she received it in a hospitalization months prior to this event. Ceftriaxone was also ruled out because the patient denied any adverse events when given to her years ago. Given her previous sulfa-drug allergy history, we believed torsemide was the causative agent as it is a sulfa drug while spironolactone is not a sulfa drug. Considering our patient’s severe liver disease, it was concluded that the possible culprit in our case was torsemide as it has hepatic metabolism in 80% of people via the CYP2C9 pathway, which is the most abundant CYP2C subfamily enzyme in the liver [12,13,14]. Torsemide undergoes extensive hepatic metabolism by tightly binding to plasma proteins such as albumin [15,16]. It is important to note that furosemide undergoes 85% renal metabolism, which further supports that furosemide was not our causative agent [15]. CYP2C9 gene polymorphisms can result in decreased enzyme activity. In fact, CYP2C9*3 variant carriers have a greatly reduced torsemide clearance [14]. It is possible our patient might have had this variant. Therefore, torsemide should be used with caution in patients with known hypersensitivity to sulfa drugs and patients with hepatic disease. Torsemide should be used concomitantly with either aldosterone antagonists or potassium-sparing agents to prevent hypokalemia and metabolic alkalosis [16]. ​​

Overall, treatment for TEN requires supportive care as well as the discontinuation of the offending agent. TEN patients often require the burn unit or intensive unit care for aggressive fluid and electrolyte management along with pain control. The most common and serious complications of TEN are organ failure and sepsis. Like with our patient, plasmapheresis is utilized to remove drug metabolites and other cytotoxic mediators from the circulation [17]. There have been other studies showing the use of biologics like etanercept or Enbrel [18]. 

## Conclusions

This report details a case of TEN in a patient with an eight-day history of torsemide use. While reports of TEN secondary to antibacterial sulfa-containing drugs have been well reported, non-antibacterial sulfa-containing drugs have been less commonly reported, and specifically torsemide-induced SJS/TEN is rare. We hope that this case demonstrates the importance of recognizing the severe side effects that medications can have, recognizing medication-induced dermatologic reactions, the importance of prompt discontinuation of the offending agent, involving a multidisciplinary team in the management of SJS/TEN patients, and having a treatment approach of patients with SJS/TEN. 
